# Monitoring and Targeted Regulation of Oxygen Metabolism in Pediatric Sepsis: Current Paradigms and Future Perspectives

**DOI:** 10.3390/ijms27104454

**Published:** 2026-05-15

**Authors:** Hong Zheng, Lijun Guan, Yiyao Bao

**Affiliations:** Department of Surgical Intensive Care Unit, National Clinical Research Center for Children and Adolescents’ Health and Diseases, Children’s Hospital, Zhejiang University School of Medicine, Hangzhou 310052, China; 6510096@zju.edu.cn (H.Z.); 6513035@zju.edu.cn (L.G.)

**Keywords:** pediatric sepsis, oxygen metabolism, lactate, tissue perfusion, microcirculation, therapeutic strategies

## Abstract

Pediatric sepsis is a life-threatening systemic infectious response syndrome. Its core pathophysiological process involves a systemic imbalance between oxygen delivery and demand, coupled with cellular energy metabolism dysfunction, which collectively contribute to high mortality rates. Parameters of oxygen metabolism serve as critical indicators reflecting tissue perfusion and cellular oxygen utilization. Consequently, these parameters hold significant value for the early identification, severity stratification, therapeutic guidance, and prognostic evaluation of pediatric sepsis. This review systematically elucidates the pathophysiological mechanisms underlying oxygen metabolism disorders in pediatric sepsis. Furthermore, it highlights the current clinical applications and significance of key monitoring indices, including blood lactate, central venous oxygen saturation, oxygen delivery, and oxygen consumption. By integrating recent research advancements, this paper also explores therapeutic strategies aimed at optimizing oxygen metabolism, such as blood purification, microcirculation-targeted therapies, and extracorporeal membrane oxygenation. Finally, we provide future perspectives on emerging biomarkers and metabolomic approaches, aiming to establish a theoretical foundation for the optimized clinical management of pediatric sepsis.

## 1. Introduction

Pediatric sepsis remains a leading cause of morbidity and mortality in pediatric intensive care units worldwide, fundamentally characterized by a dysregulated host response to infection that culminates in life-threatening organ dysfunction [1]. During this pathophysiological process, the confluence of systemic inflammatory responses, endothelial injury, and microcirculatory dysfunction precipitates a critical imbalance between tissue oxygen delivery and consumption, thereby triggering cellular hypoxia and profound energy metabolism crises [2]. The state of oxygen metabolism serves as the central nexus bridging macroscopic hemodynamics and microscopic cellular function; consequently, its precise monitoring and effective regulation are paramount for improving clinical outcomes in pediatric patients [3].

Recent advances in monitoring technologies and sepsis pathophysiology have increased interest in oxygen-metabolism parameters in clinical practice [4]. Dynamic indices, such as lactate trends or lactate clearance, may provide more useful prognostic information than isolated measurements [4]. However, the prognostic value of traditional targets such as central venous oxygen saturation (ScvO2) remains debated, and emerging metabolic biomarkers are being explored as complementary tools [5]. Therapeutic strategies such as blood purification, microcirculatory optimization, and advanced supportive therapies may influence oxygen delivery or utilization, but their clinical value in pediatric sepsis depends on the strength of supporting evidence [6].

Assessment of end-organ perfusion and pediatric microcirculation further supports the need to interpret oxygen-metabolism abnormalities within a pediatric-specific framework [7].

Therefore, this review aims to examine oxygen-metabolism dysfunction in pediatric sepsis with a focus on clinically relevant monitoring parameters, pediatric-specific physiological considerations, and therapeutic strategies that directly influence oxygen delivery, oxygen utilization, or microcirculatory perfusion. Emerging approaches, including AI-assisted prediction, multi-omics profiling, SIRT-related pathways, molecular hydrogen, and therapeutic hypothermia, are discussed only where they are directly connected to oxygen-metabolism assessment or modulation. Throughout the review, we distinguish pediatric clinical evidence from adult-extrapolated evidence, observational associations, preclinical findings, and theoretical mechanisms, particularly when discussing therapeutic implications. Because pediatric sepsis spans developmentally distinct populations, this review treats neonates, infants, children, and adolescents as physiologically non-equivalent groups whenever evidence allows, rather than assuming a single pediatric response pattern.

## 2. Pathophysiological Mechanisms of Oxygen Metabolism Dysfunction

### 2.1. Macro-Hemodynamic Uncoupling and Microcirculatory Heterogeneity

**Figure 1:**
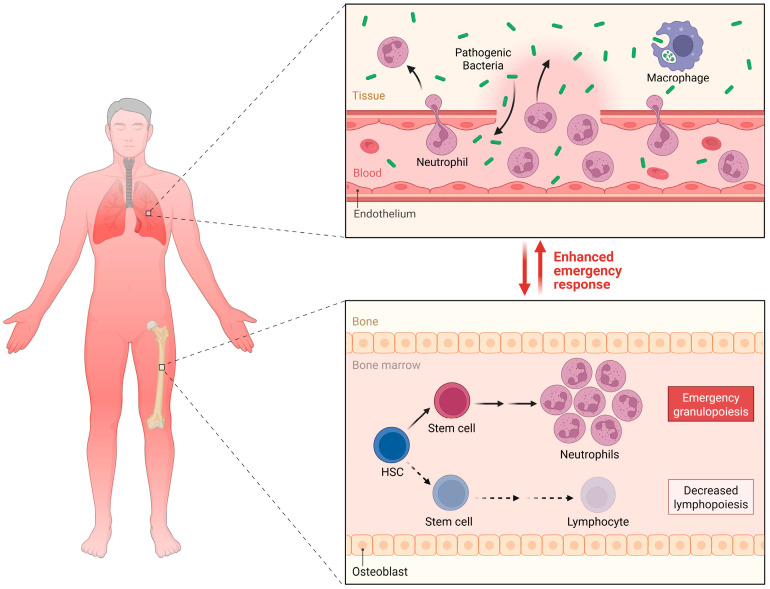
The multi-level cascade of oxygen metabolism dysfunction in pediatric sepsis.

In the pathophysiological progression of pediatric sepsis, the systemic imbalance between oxygen supply and demand, coupled with microcirculatory dysfunction, constitutes the core mechanism precipitating tissue hypoperfusion and multiple organ failure [8,9,10]. Although some children may exhibit a hyperdynamic state with increased cardiac output during the early phase of sepsis, this pattern is not uniform across pediatric age groups. Neonates and young infants have limited myocardial reserve, immature autonomic and vascular tone regulation, and relatively high baseline oxygen consumption, which may narrow their tolerance for impaired oxygen delivery. Older children and adolescents may more closely resemble adult hemodynamic phenotypes, but developmental differences in cardiac reserve, vascular responsiveness, and metabolic demand still limit direct extrapolation from adult thresholds. Across all age groups, apparent normalization of macroscopic hemodynamics may mask persistent microvascular dysfunction. These microcirculatory derangements primarily manifest as severe vasodilation, abnormal blood flow distribution, and the pathological opening of arteriovenous shunts. The collective consequence is a significant reduction in the blood flow effectively perfusing the capillary beds, thereby leaving tissue cells in a state of hypoxia even when systemic oxygen delivery appears adequate [6], as shown in Figure 1.

The heterogeneity of microcirculatory blood flow is a hallmark alteration in sepsis. This phenomenon is characterized by sluggish or completely absent flow in certain capillaries, juxtaposed with normal or hyperemic flow in adjacent areas. This profound perfusion inequality severely impairs overall tissue oxygenation, elucidating why organ dysfunction may continue to progress despite the restoration of macroscopic hemodynamic parameters [11,12,13]. Concurrently, endothelial cell injury and the shedding of its protective surface glycocalyx serve as another critical mechanism exacerbating microcirculatory impairment. The degradation of the glycocalyx not only compromises the vascular barrier function, leading to a marked increase in capillary permeability, but also precipitates severe tissue edema [14,15,16]. The subsequent accumulation of interstitial fluid increases the diffusion distance for oxygen from the capillaries to the cellular mitochondria, further impeding effective oxygen transport and utilization, and thereby intensifying hypoxia at the cellular level.

Consequently, targeted assessment and modulation of the microcirculation have emerged as important strategies for improving tissue perfusion and oxygenation in pediatric sepsis. Potential approaches include the use of balanced crystalloids during fluid resuscitation to reduce hyperchloremic acidosis and endothelial dysfunction, as well as the correction of hypoalbuminemia to support glycocalyx stability and preserve vascular integrity [6], as shown in Table 1.

### 2.2. Mitochondrial Dysfunction and the “Cytopathic Hypoxia” Paradigm

Pediatric sepsis can also impair oxygen metabolism at the cellular level. In addition to macro-hemodynamic and microcirculatory abnormalities, inflammatory signaling may disrupt mitochondrial function and contribute to “cytopathic hypoxia” [6]. Mitochondrial injury can suppress electron transport chain activity and impair oxidative phosphorylation, limiting the ability of cells to generate adenosine triphosphate (ATP) even when oxygen delivery has been partially restored. The direct consequence is that, even if tissue oxygen delivery is adequately restored through therapeutic interventions, cells remain incapable of effectively utilizing oxygen to synthesize adenosine triphosphate (ATP), culminating in a profound deficit in energy production [17].

This critical defect in cellular oxygen utilization compels cells to shift from highly efficient aerobic metabolism to inefficient anaerobic glycolysis to acquire minimal energy. A prominent byproduct of this anaerobic glycolysis is the substantial generation of lactate; therefore, the development of hyperlactatemia in sepsis is centrally driven by this intrinsic cellular metabolic shift, rather than being solely attributable to inadequate tissue hypoperfusion [17]. Furthermore, intracellular metabolic derangements are not confined exclusively to mitochondria. The functions of other organelles, such as peroxisomes, are concurrently compromised. This interference disrupts normal fatty acid metabolism and attenuates the cellular capacity to scavenge reactive oxygen species (ROS). The subsequent accumulation of ROS exacerbates oxidative stress, causing further damage to mitochondrial structure and function. This dynamic creates a vicious cycle that continuously worsens cellular metabolic imbalance and the underlying energy crisis [18]. Consequently, comprehending and intervening in this cellular-level metabolic dysfunction is of paramount importance for overcoming current bottlenecks in pediatric sepsis treatment and improving overall patient prognosis.

## 3. Multimodal Monitoring of Oxygen Metabolism

### 3.1. Traditional Surrogate Markers: Lactate Kinetics and ScvO2

Blood lactate levels have long served as the classical surrogate marker for tissue hypoperfusion and cellular anaerobic metabolism, occupying a central position in the clinical assessment of pediatric sepsis. International guidelines consistently recommend lactate measurement as a frontline diagnostic modality [19]. However, the clinical interpretation of lactate necessitates a nuanced approach. Extensive evidence indicates that the dynamic evolution of lactate (lactate clearance) provides superior prognostic accuracy compared to isolated absolute values. Specifically, a prospective observational study demonstrated that 24 h lactate clearance is an optimal early predictor of pediatric sepsis mortality, yielding an area under the curve (AUC) of 0.958 [20]. Furthermore, persistent hyperlactatemia, such as an initial pediatric intensive care unit (PICU) admission level exceeding 5 mmol/L, is significantly associated with elevated mortality and the progression of organ failure [21]. The lactate-to-albumin ratio (LAR) has also emerged as a robust composite index; an early LAR greater than 0.5 correlates with higher mortality, and its elevation in septic children is significantly linked to microvascular flow abnormalities and reduced capillary density [22]. Notably, contemporary research emphasizes the critical need to distinguish between “stress-induced” and “ischemic” lactate elevations. For instance, in pediatric patients with diabetic ketoacidosis, hyperlactatemia does not invariably signify a poor prognosis, underscoring physiological mechanisms distinct from those in sepsis [23]. Therefore, monitoring continuous lactate kinetics and trends is substantially more effective for predicting clinical outcomes than relying on single cross-sectional readings [4].

ScvO2 reflects the global balance between systemic oxygen delivery (DO2) and oxygen consumption (VO2), but its interpretation differs between adults and children. Pediatric ScvO2 values may be influenced by age-dependent oxygen consumption, hemoglobin concentration, cardiac output distribution, sedation, congenital or chronic disease, and the capacity for oxygen extraction. A decreased ScvO2 typically indicates inadequate oxygen delivery or excessive metabolic consumption, whereas a normal or elevated ScvO2 in the presence of hyperlactatemia often suggests microcirculatory shunting or underlying mitochondrial dysfunction. It is crucial to recognize that the physiological baseline of ScvO2 may require adjustment in specific pediatric subpopulations. A prospective analysis of clinically stable children with cancer revealed a mean predicted ScvO2 of 0.67 (95% confidence interval: 0.56–0.78), which is significantly lower than the conventional normal range of 0.70 to 0.80 and closely correlates with baseline hemoglobin concentrations [24]. The precise quantification of oxygen delivery and consumption parameters is instrumental in guiding targeted therapies. Retrospective data indicate that integrated blood purification modalities can significantly optimize oxygen metabolism in severe pediatric sepsis; these treatments effectively reduce the heart rate, stroke volume, blood lactate, oxygen consumption, and oxygen extraction ratio, while concurrently increasing systolic blood pressure and central venous oxygen tension [25]. Accordingly, ScvO2 should be interpreted as part of a multimodal pediatric assessment rather than as a fixed adult-derived therapeutic target.

The integration of these traditional markers into a multimodal assessment framework is imperative to avoid clinical misjudgments. The international consensus on the Phoenix Sepsis Score incorporates cardiovascular dysfunction criteria, explicitly including a blood lactate threshold exceeding 5 mmol/L [26]. When integrating these indices, clinical phenotypes become clearer. For example, a clinical scenario characterized by severe hyperlactatemia but a normal ScvO2 implies that the primary pathology resides in microcirculatory flow maldistribution or impaired mitochondrial oxygen utilization, rather than a macroscopic cardiac output deficit [27]. In such instances, blindly escalating macroscopic cardiac output may prove not only ineffective but potentially deleterious; thus, the therapeutic focus must pivot toward microcirculatory optimization and targeted cellular metabolic interventions [8], as shown in Table 2.

### 3.2. Direct Microcirculatory Assessment: Sublingual Imaging and Bedside Modalities

While macroscopic hemodynamic parameters provide foundational data, they frequently fail to capture the complexities of tissue-level perfusion. Consequently, direct microcirculatory assessment has emerged as a crucial component of multimodal monitoring. As a fundamental physical examination technique for evaluating distal perfusion, capillary refill time has been proven to correlate significantly with the prognosis of sepsis; however, its manual measurement is inherently subject to substantial inter-observer variability. Therefore, the development of objective and rapid bedside assessment technologies is of paramount importance for achieving precision resuscitation [28].

To bridge this gap, handheld incident dark-field imaging technology provides a highly effective tool for the real-time, bedside assessment of microcirculatory status. This advanced technique enables the direct visualization of the sublingual microcirculation, thereby facilitating the direct observation of the terminal phase of oxygen delivery. A specialized study investigating severe trauma demonstrated that sublingual microvascular perfusion, directly measured via handheld incident dark-field microscopy, yielded calculated total vessel densities and De Backer scores that significantly correlated with serum lactate levels. Conversely, these precise microcirculatory parameters exhibited weak correlations with indirect, systemic perfusion markers, such as mean arterial pressure and heart rate [29]. This critical finding indicates that the direct visualization of the microcirculation more accurately reflects the state of oxygen metabolism at the tissue level. Particularly in severe conditions like sepsis, indirect macroscopic indicators may completely fail to genuinely represent the underlying microvascular dysfunction.

Nevertheless, although direct microcirculatory imaging has substantial potential, its standardized application in children remains challenging. Neonates and infants have smaller oral and sublingual surfaces, fragile mucosa, limited cooperation, and frequent movement, which may reduce image quality and reproducibility. Older children and adolescents may allow more reliable image acquisition, but age-related differences in vessel density, perfusion reserve, disease phenotype, sedation status, and vasoactive exposure may still affect interpretation. Therefore, pediatric microcirculatory monitoring requires age-stratified feasibility data, reference ranges, and outcome-linked thresholds before it can be routinely used to guide therapy. This inherent heterogeneity necessitates the rigorous establishment of normal reference values and pathological thresholds tailored to distinct pediatric age groups. Furthermore, the accurate interpretation of microcirculatory images requires specialized professional training. Most importantly, the definitive correlation between these microcirculatory imaging parameters and robust clinical outcomes in pediatric sepsis remains to be rigorously validated through large-scale, prospective clinical trials.

### 3.3. Emerging Oxygen-Metabolism Biomarkers and Selected Omics-Based Signals

Beyond traditional monitoring parameters, emerging metabolic markers and multi-omics signatures are demonstrating profound diagnostic and prognostic potential in pediatric sepsis. The respiratory quotient (RQ), which reflects systemic substrate utilization, has garnered attention in critical care. In pediatric patients with septic shock, an elevated RQ serves as an independent risk factor for mortality, a phenomenon likely associated with a hypercatabolic state and futile energy cycling. Furthermore, transcriptomic analyses have paved the way for discovering novel molecular targets. For instance, peroxisome-related genes, such as EPHX2 and IDH1, exhibit differential expression in septic children, and their pathway activities are intricately linked to immune-inflammatory cascades. Concurrently, the dysbiosis of the gut microbiome correlates significantly with clinical indices; specifically, Shannon diversity is significantly associated with leukocyte counts, serum lactate, and the length of stay in the pediatric intensive care unit [30]. In addition to genomic data, heparin-binding protein has emerged as a robust protein biomarker. A multicenter prospective study demonstrated that elevated levels of heparin-binding protein significantly enhance the diagnostic capacity for severe pediatric sepsis when integrated with procalcitonin and lactate models, while its dynamic alterations are independently associated with hospital mortality [31].

The application of artificial intelligence and machine learning to multi-omics data provides a powerful perspective for deciphering the heterogeneity of oxygen metabolism disorders. By analyzing peripheral blood transcriptomes across multi-center sepsis cohorts, machine learning algorithms have identified key differentially expressed genes associated with ferroptosis. Notably, the MAP1LC3B gene exhibits exceptional predictive capability in pediatric populations, underscoring its pivotal role in regulating mitochondrial reactive oxygen species and cellular energy metabolism [32].

Metabolomic profiling may help characterize energy dysregulation in sepsis. Mitochondrial dysfunction contributes to multiorgan failure, and excessive reactive oxygen and nitrogen species can damage respiratory chain enzymes and impair cellular metabolism [33]. However, the translation of metabolomic findings into bedside pediatric sepsis management remains limited. Consequently, mapping the dynamic metabolomic fingerprints of carbohydrates, lipids, and amino acids can facilitate ultra-early targeted interventions. Targeting specific metabolic pathways, such as the Sirtuin (SIRT) family, offers a promising strategy to modulate cellular inflammation, improve mitochondrial function, and restore energy balance [34]. Moreover, agents like molecular hydrogen exhibit therapeutic potential by scavenging excessive reactive oxygen species and regulating mitochondrial energy metabolism, further highlighting the shift toward targeted metabolic resuscitation [35], as shown in Table 3.

## 4. Oxygen Metabolism-Targeted Therapeutic Strategies

When interpreting oxygen-metabolism-targeted therapies in pediatric sepsis, the strength and source of evidence must be explicitly considered. Interventions such as early antimicrobial therapy, source control, oxygenation support, fluid resuscitation, and vasoactive therapy are embedded in current pediatric sepsis care, although optimal targets may vary by age and hemodynamic phenotype. In contrast, microcirculation-directed interventions, albumin replacement for glycocalyx protection, FFP for endothelial repair, vasodilator-based strategies, extracorporeal support for metabolic modulation, therapeutic hypothermia, molecular hydrogen, and omics-guided therapies are supported by varying levels of evidence, ranging from pediatric observational studies to adult extrapolation, animal experiments, or mechanistic rationale. Therefore, these approaches should be interpreted as evidence-dependent and context-specific rather than uniformly established pediatric therapies.

### 4.1. Precision Resuscitation and Macro-Hemodynamic Optimization

**Figure 2:**
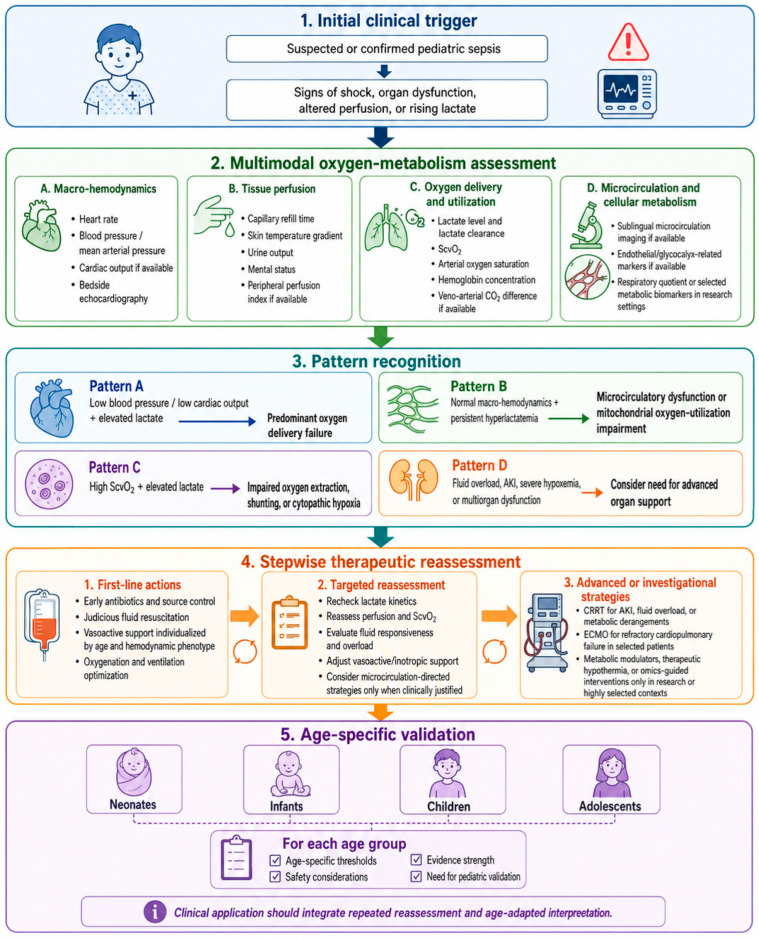
Proposed multimodal assessment and stepwise management framework for oxygen-metabolism dysfunction in pediatric sepsis. The framework emphasizes integrated interpretation of macro-hemodynamic variables, tissue perfusion signs, oxygen delivery and utilization markers, and microcirculatory or metabolic indicators. Abnormal patterns should prompt repeated reassessment rather than isolated target correction. Therapeutic responses should be individualized according to age group, hemodynamic phenotype, disease severity, and evidence strength. Advanced strategies such as CRRT, ECMO, therapeutic hypothermia, metabolic modulators, or omics-guided interventions should be considered only in selected clinical or research contexts when pediatric evidence is limited.

The traditional early goal-directed therapy (EGDT) paradigm, which was largely developed from adult sepsis resuscitation and included targets such as ScvO2 greater than 70%, has faced increasing scrutiny when applied to pediatric sepsis. Recent evidence indicates that resuscitation protocols based solely on isolated, static hemodynamic parameters have lost their comparative advantage over conventional therapies [36]. Although the early recognition and prompt initiation of antimicrobial and fluid therapies remain unequivocally essential, the rigid targets of traditional EGDT are highly controversial in pediatric cohorts [37]. Current resuscitation strategies increasingly emphasize dynamic reassessment and individualized treatment guided by multimodal monitoring. Parameters such as cardiac output, systemic vascular resistance, lactate kinetics, perfusion signs, and bedside ultrasound may provide complementary information when used together, but no single target is sufficient for all pediatric patients. Modern resuscitation endpoints now require integrated assessment of macro-hemodynamics, tissue perfusion, microcirculatory status, and cellular oxygen utilization. In this context, mean arterial pressure, lactate kinetics, ScvO2, capillary refill time, bedside ultrasonography, and, where available, microcirculatory or metabolic monitoring should be interpreted together rather than in isolation. Figure 2 summarizes a proposed pediatric sepsis oxygen-metabolism assessment and treatment framework, linking abnormal monitoring patterns to stepwise therapeutic reassessment. Age group should be considered when selecting resuscitation targets. Neonates and young infants may have limited ability to increase stroke volume and may rely more heavily on heart rate to maintain cardiac output, making them vulnerable to both inadequate preload and fluid overload. Older children and adolescents may tolerate broader hemodynamic variation, but myocardial dysfunction, vasoplegia, and distributive shock can still produce distinct oxygen-delivery phenotypes. Consequently, fluid administration, vasoactive selection, and reassessment intervals should be individualized according to developmental physiology, cardiac function, perfusion response, and risk of fluid accumulation.

In the context of fluid resuscitation, the strategic administration of balanced crystalloids is gaining substantial prominence. This approach specifically aims to minimize the incidence of hyperchloremic acidosis commonly associated with unbalanced solutions, thereby mitigating endothelial dysfunction and promoting the recovery of microcirculatory oxygen metabolism [6]. Because microcirculatory dysfunction is a primary determinant of tissue hypoperfusion and multiorgan failure, utilizing balanced solutions effectively attenuates hyperchloremia, metabolic acidosis, and subsequent inflammatory activation at the endothelial interface [6]. Furthermore, highly individualized hemodynamic support is uniquely critical for specific high-risk pediatric populations, such as those undergoing hematopoietic stem cell transplantation [38]. Implementing these standardized yet personalized resuscitation protocols also requires careful consideration of sociodemographic variables; studies demonstrate that ethnic disparities independently correlate with pediatric intensive care unit admission risks and influence adherence to EGDT pathways [39]. Ultimately, the integration of advanced artificial intelligence tools with multimodal physiological data is imperative for realizing truly individualized, dynamic resuscitation, thereby fundamentally improving oxygen metabolism and clinical outcomes in pediatric sepsis.

### 4.2. Microcirculatory and Endothelial Protection: Albumin, Vasoactive Agents, and FFP

In pediatric sepsis, microcirculatory dysfunction operates as a pivotal determinant of tissue hypoperfusion and multiorgan failure, with its pathogenesis intricately linked to endothelial glycocalyx degradation, heightened capillary permeability, and pronounced flow heterogeneity [9,10]. Consequently, therapeutic strategies targeting the microcirculation are critical for optimizing clinical outcomes. Hypoalbuminemia is clinically relevant in this context, but the evidence should be interpreted carefully. Pediatric observational studies indicate that hypoalbuminemia in septic children is associated with microvascular alterations, increased vascular permeability, and endothelial glycocalyx shedding [40]. These findings support albumin as a potential marker of endothelial and microcirculatory dysfunction, whereas the therapeutic benefit of albumin replacement for improving oxygen metabolism or clinical outcomes remains insufficiently validated in pediatric sepsis. Specifically, patients with hypoalbuminemia exhibit an elevated perfused boundary region, reflecting diminished glycocalyx thickness and compromised function, accompanied by a compensatory recruitment of small capillaries measuring 4 to 6 micrometers in diameter [40]. This structural degradation destabilizes the vascular barrier, thereby exacerbating capillary leakage. Therefore, targeted albumin replacement to stabilize the endothelial glycocalyx theoretically ameliorates microvascular perfusion and tissue oxygenation. Furthermore, an elevated lactate-to-albumin ratio correlates strongly with abnormal microvascular blood flow and decreased small capillary density, underscoring the vital role of albumin in maintaining microcirculatory homeostasis [22].

Inotropes and vasodilator-based strategies may influence microcirculatory perfusion, but the pediatric evidence base remains limited. Their rationale is largely derived from physiological principles, adult critical-care experience, and selected experimental or observational studies. By augmenting myocardial contractility, modifying vascular tone, or reducing afterload in selected hemodynamic phenotypes, these agents may improve convective and diffusive components of tissue oxygen delivery; however, the net clinical benefit and optimal patient selection in pediatric sepsis remain uncertain [6]. While the administration of these vasoactive medications has demonstrated favorable regulatory effects on capillary perfusion and inflammatory responses in septic shock, their definitive net clinical benefit in pediatric cohorts necessitates further rigorous investigation. The integration of automated sublingual microcirculation analysis has proven instrumental in evaluating treatment responses; for instance, interventions promoting systemic vasodilation and increased cardiac output have been shown to independently improve the diffusive and convective capacities of the microcirculation [41]. This highlights the promising potential of individualized pharmacological strategies designed to resolve the dissociation between macrovascular hemodynamics and microcirculatory perfusion, a phenomenon often termed “hemodynamic incoherence.” The Resuscitation, Equilibrium, and De-escalation (RED) strategy further emphasizes the necessity of tailored, phased hemodynamic support, carefully titrating the initiation and duration of vasoactive medications based on dynamic patient profiles [42].

Fresh frozen plasma (FFP) has also been proposed as a potential endothelial or glycocalyx-directed intervention, but this concept remains mainly mechanistic. The theoretical rationale is that plasma components might help replenish glycocalyx constituents and support endothelial homeostasis; however, pediatric sepsis data are insufficient to support routine FFP transfusion for the purpose of improving microcirculatory oxygen metabolism. Because the intact endothelial glycocalyx is the primary determinant of vascular permeability, its degradation during sepsis directly provokes profound capillary leakage [43]. Consequently, FFP transfusion is hypothesized to be a viable microcirculation-targeted therapy aimed at repairing the glycocalyx and re-establishing endothelial integrity. However, despite a compelling physiological rationale, its routine incorporation into pediatric sepsis management still lacks validation from high-level clinical trials [6]. Current research predominantly focuses on correlating microcirculatory monitoring parameters with prognostic outcomes. For example, a prolonged capillary refill time is significantly associated with extensive glycocalyx degradation and a failure to recruit small capillaries, while persistent hemodynamic incoherence consistently predicts adverse clinical trajectories [44]. These findings unequivocally underscore the prognostic value of microcirculatory monitoring, yet they do not firmly substantiate the efficacy of specific interventions like FFP. Extensive prospective studies are urgently required to validate the impact of these microcirculation-targeted therapies on pediatric morbidity and mortality, facilitating their integration into precision management protocols [6,43], as shown in Table 4.

### 4.3. Advanced Extracorporeal Support and Metabolic Modulation: ECMO, CRRT, and Therapeutic Hypothermia

Advanced extracorporeal therapies should be interpreted according to their primary clinical indication and evidence source. In pediatric sepsis, ECMO is primarily considered for selected patients with refractory cardiopulmonary failure, and CRRT is primarily used for acute kidney injury, fluid overload, severe electrolyte or acid-base disturbances, and multiorgan dysfunction. Their relevance to oxygen metabolism is often indirect, through restoration of oxygenation, hemodynamic stabilization, fluid balance, or metabolic homeostasis. By contrast, using ECMO, CRRT, or therapeutic hypothermia specifically as oxygen-metabolism-targeted interventions remains less well established and requires cautious interpretation.

Venovenous extracorporeal membrane oxygenation (VV-ECMO) operates as an advanced extracorporeal life support modality, playing a paramount role in the management of pediatric sepsis, particularly when complicated by severe pediatric acute respiratory distress syndrome (PARDS). By bypassing the compromised pulmonary system, VV-ECMO facilitates highly efficient, direct gas exchange, rapidly rectifying life-threatening hypoxemia and hypercapnia [45]. This intervention profoundly diminishes the pulmonary oxygen consumption burden and establishes a conducive environment for systemic oxygen metabolism recovery. Evidence confirms that the initiation of VV-ECMO yields immediate and sustained improvements in oxygenation; for instance, successfully weaned patients with burn-induced ARDS exhibited significantly elevated arterial oxygen tension (PaO2) and oxygen saturation (SaO2), alongside a reduction in the fraction of inspired oxygen (FiO2) to below 50% and an enhanced oxygenation index exceeding 200 mmHg [45]. This rapid metabolic optimization directly translates to hemodynamic stability, quantitatively reflected by a reduction in the vasoactive-inotropic score (VIS) [46]. Furthermore, a retrospective pediatric analysis identified high PELOD-2 scores, the presence of sepsis, and the requirement for continuous renal replacement therapy (CRRT) as critical predictors of in-hospital mortality during ECMO support, underscoring the inherent challenges and the indispensability of this technology in stabilizing the most critically ill cohorts [46,47].

In the management of severe pediatric sepsis complicated by acute kidney injury, fluid overload, or multiorgan dysfunction, CRRT is primarily used as renal and fluid-supportive therapy. Its potential relevance to oxygen metabolism is indirect: by controlling fluid balance, correcting acid-base and electrolyte disturbances, and supporting metabolic homeostasis, CRRT may help reduce secondary physiological stress in selected critically ill children. However, evidence that CRRT directly improves tissue oxygen utilization or reverses cellular metabolic dysfunction remains limited.

A large-scale, international multicenter WE-ROCK retrospective analysis evaluated factors associated with successful liberation from CRRT in children and young adults [48]. This study should be interpreted as evidence regarding CRRT course and liberation outcomes, rather than as proof of a direct oxygen-metabolism benefit. Accordingly, CRRT-related conclusions in pediatric sepsis should be framed cautiously and should account for disease severity, organ dysfunction, vasoactive support, and the frequent coexistence of ECMO or other advanced life-support modalities [49,50,51].

Therapeutic hypothermia, or targeted temperature management, has a plausible mechanistic relationship with oxygen metabolism because lowering body temperature can reduce metabolic demand and oxygen consumption. Nevertheless, pediatric sepsis-specific evidence remains insufficient, and current data do not establish that therapeutic hypothermia improves survival, reduces vasoactive-inotropic support, shortens mechanical ventilation, or improves long-term neurodevelopmental outcomes. Therefore, it should be presented as an investigational metabolic-modulation strategy that requires prospective pediatric validation, rather than as a routine adjunctive therapy.

## 5. Pediatric-Specific Challenges and Future Directions

### 5.1. Bridging the Pediatric Evidence Gap: Age-Specific Physiology and the Lack of High-Level RCTs

The monitoring and targeted regulation of oxygen metabolism in pediatric sepsis encounter significant age-specific challenges, primarily stemming from the fundamental physiological differences between pediatric patients (particularly neonates and infants) and adults. Regarding the cardiovascular system, children possess immature myocardial reserves and underdeveloped vascular tone regulatory mechanisms. This physiological immaturity dictates that their responses to volume expansion and vasoactive agents diverge substantially from those of adults; consequently, normal reference values and clinical intervention thresholds for oxygen metabolism parameters (such as ScvO2 and the veno-arterial carbon dioxide tension difference) derived from adult cohorts cannot be directly extrapolated to pediatric populations [52]. For example, neonates exhibit inherently higher baseline oxygen consumption rates and unique cardiac output distributions, implying that their “normal” ScvO2 range may be significantly higher than that of older children or adults. Therefore, establishing and robustly validating reference ranges for oxygen metabolism parameters tailored to specific age groups and weight brackets constitutes a critical prerequisite for precision therapy; however, high-quality data in this specific domain remain conspicuously scarce.

A more profound challenge resides in the stark dearth of high-level evidence supporting advanced interventions in pediatric cohorts. The dosages, efficacies, and safety profiles of numerous vasoactive agents, inotropes, and prospective microcirculation-targeted therapies currently utilized in clinical practice are predominantly extrapolated from adult studies, small-scale observational research, or expert consensus, rather than rigorously designed randomized controlled trials (RCTs) [27]. This reliance on extrapolation inherently breeds substantial uncertainty in clinical decision-making. Although emerging technologies, such as artificial intelligence-integrated wearable devices, offer novel possibilities for the continuous monitoring of physiological parameters, their application in pediatrics is largely confined to small-scale, single-center feasibility studies. These preliminary investigations primarily focus on signal validity and device tolerability, consistently failing to robustly demonstrate favorable impacts on hard clinical endpoints, including mortality, readmission rates, or long-term neurodevelopmental recovery [27,53].

Furthermore, several practical and ethical barriers limit the translation of novel monitoring technologies into routine pediatric practice. Device design must account for age-dependent anatomical and dermatological differences, including smaller limb circumference, limited vascular access sites, immature or fragile skin in neonates, variable subcutaneous tissue thickness, edema, peripheral vasoconstriction, and differences in skin pigmentation. These factors may affect sensor contact, optical signal penetration, motion tolerance, calibration accuracy, and the reliability of perfusion-related measurements. Additional challenges include frequent movement, crying, probe displacement, limited cooperation in younger children, data privacy requirements for wearable or AI-enabled devices, and the lack of pediatric-specific labeling or regulatory approval for many diagnostic technologies [27]. Future studies should therefore evaluate monitoring devices separately across neonates, infants, children, and adolescents, and should report age-specific feasibility, signal quality, safety, and clinical validity before these technologies are incorporated into pediatric sepsis pathways, as shown in Table 5.

### 5.2. Focused Future Directions: AI-Assisted Monitoring and Pediatric Validation

AI-assisted monitoring may support pediatric sepsis care by integrating continuous physiological signals, such as electrocardiography, photoplethysmography, pulse oximetry, temperature, perfusion indices, and movement data. In the context of oxygen metabolism, the most clinically relevant applications are early detection of perfusion deterioration, identification of abnormal lactate or ScvO2 trajectories, recognition of microcirculatory risk patterns, and support for individualized reassessment after resuscitation. However, these applications remain dependent on high-quality pediatric datasets, external validation, interpretable model outputs, and careful assessment of bias across age groups and care settings [27].

Therefore, AI should currently be presented as a supportive tool for risk stratification and monitoring rather than as an autonomous treatment-directing system. Future studies should prioritize pediatric sepsis cohorts, age-specific model calibration, prospective validation, and clinically meaningful endpoints, such as earlier recognition of shock progression, reduced time to treatment adjustment, organ dysfunction, and survival. This focused approach would better align AI development with the practical goal of improving oxygen delivery, tissue perfusion, and cellular oxygen utilization in children, as shown in Table 6.

## 6. Conclusions

Management of pediatric sepsis increasingly requires attention not only to macro-hemodynamic resuscitation but also to tissue perfusion, microcirculatory function, and cellular oxygen utilization. Traditional indices, including blood lactate and central venous oxygen saturation, provide macroscopic clues regarding the imbalance between oxygen delivery and consumption, whereas emerging metabolomic and transcriptomic biomarkers, alongside the respiratory quotient, elucidate metabolic reprogramming and dysfunction at the cellular and mitochondrial levels. Together, these parameters constitute a multidimensional assessment framework spanning from the systemic circulation to the intracellular microenvironment. This paradigm necessitates that clinicians seamlessly integrate information across disparate levels. It is imperative to remain vigilant regarding the systemic oxygen debt indicated by elevated lactate, while simultaneously acknowledging that normolactatemia may mask regional microcirculatory derangements or mitochondrial suppression, thereby circumventing the clinical pitfalls of isolated parameter interpretation.

The ultimate common pathway of contemporary therapeutic strategies, such as goal-directed fluid resuscitation, microcirculation-optimizing pharmacological or physical interventions, integrated blood purification, and advanced life support modalities like VV-ECMO, is the enhancement of tissue oxygen delivery and utilization efficiency. However, the efficacy of these interventions exhibits pronounced heterogeneity across diverse pediatric cohorts and distinct disease stages. For instance, while therapeutic hypothermia may confer benefits by reducing the metabolic rate in specific subpopulations, it simultaneously risks inducing immunosuppression. Consequently, the critical challenge for the future lies in actualizing truly individualized therapy. Overcoming this challenge relies on two fundamental breakthroughs. Technologically, there is an urgent need to develop non-invasive, bedside, real-time monitoring tools for microcirculatory and cellular metabolism to visualize previously imperceptible metabolic disturbances. Cognitively and computationally, it is essential to leverage multi-omics technologies and artificial intelligence to rigorously decipher the vast diversity of metabolic phenotypes in pediatric sepsis, enabling the precise identification of subpopulations primed to benefit from specific interventions, such as mitochondrial protectants or metabolic modulators.

Looking ahead, the cornerstone for advancing oxygen metabolism management in pediatric sepsis is the execution of rigorously designed, prospective clinical trials explicitly tailored to pediatric populations. Only through the generation of high-quality evidence can the definitive value of various oxygen metabolism-directed strategies (e.g., specific blood purification modalities and microcirculation-targeted pharmacotherapies) be established regarding their capacity to reduce mortality and improve long-term outcomes, such as neurodevelopmental recovery. The ultimate objective is to translate a profound mechanistic understanding of oxygen metabolism disorders into highly actionable clinical pathways. This transformation will catalyze the crucial leap from empirical “supportive care” to definitive “etiological correction,” thereby significantly ameliorating the clinical trajectories of this devastating critical illness. Because much of the mechanistic and interventional evidence in sepsis derives from adult cohorts, animal studies, or mixed critical-care populations, future work should avoid assuming direct transferability to children and should instead validate oxygen-metabolism targets separately in neonates, infants, children, and adolescents.

## Figures and Tables

**Table 1:** Key pathophysiological drivers of oxygen metabolism dysfunction in pediatric sepsis.

Pathophysiological Mechanism	Key Manifestations	Clinical Consequences
Macro-micro Hemodynamic Uncoupling	Vasodilation; abnormal flow distribution; opening of arteriovenous shunts.	Hypoperfusion of capillary beds despite normalized systemic cardiac output.
Microcirculatory Heterogeneity	Unequal perfusion (sluggish/absent flow adjacent to hyperemic flow).	Severe impairment of overall tissue oxygenation; progressive organ failure.
Endothelial & Glycocalyx Injury	Shedding of the protective glycocalyx; compromised vascular barrier.	Increased capillary permeability; tissue edema; increased oxygen diffusion distance.

**Table 2:** Interpretation and multimodal integration of traditional oxygen metabolism markers.

Biomarker	Clinical Indication	Prognostic Thresholds & Kinetics	Diagnostic Caveats & Multimodal Context
Blood Lactate	Global tissue hypoperfusion; cellular anaerobic glycolysis.	Initial level > 5 mmol/L indicates high mortality risk; 24 h clearance is superior to single values.	Must differentiate ischemic vs. stress-induced elevation; elevated levels with normal ScvO2 indicate microvascular or mitochondrial deficits.
ScvO2	Systemic oxygen supply and demand balance (DO2/VO2 ratio).	Normal range (typically 0.70–0.80); baseline adjustments required for specific populations (e.g., oncology patients).	Normal or high ScvO2 does not rule out localized tissue hypoxia; frequently masked by microvascular shunting.
Lactate-to-Albumin Ratio (LAR)	Composite marker of metabolic stress and vascular endothelial integrity.	Early LAR > 0.5 is strongly correlated with increased mortality and organ failure.	Elevation directly correlates with diminished capillary density and severe microcirculatory flow heterogeneity.

**Table 3:** Emerging biomarkers and multi-omics signatures in pediatric sepsis.

Modality/Biomarker	Primary Biological Pathway	Clinical and Prognostic Significance
Respiratory Quotient (RQ)	Systemic substrate utilization and energy balance.	Elevated levels indicate hypercatabolism and correlate with an increased mortality risk.
Heparin-Binding Protein	Endothelial activation and systemic inflammation.	Enhances diagnostic models for severe sepsis and independently predicts hospital mortality.
Transcriptomic Signatures (e.g., MAP1LC3B)	Mitochondrial ROS metabolism and ferroptosis regulation.	Enables machine-learning-driven prediction of disease severity and immune suppression.
Metabolomic Fingerprints (e.g., SIRT pathways)	Mitochondrial respiration and cellular oxidative stress.	Facilitates ultra-early targeted therapies to restore cellular energy equilibrium and oxygen utilization.

**Table 4:** Interventions for Microcirculatory and Endothelial Protection in Pediatric Sepsis.

Therapeutic Modality	Primary Pathophysiological Target	Proposed Mechanism of Action	Evidence Category	Current Evidence and Key Limitations
Albumin replacement	Hypoalbuminemia; endothelial glycocalyx disruption; vascular leakage	May help maintain oncotic pressure and support endothelial barrier stability; correction of hypoalbuminemia may theoretically reduce capillary leakage and improve microcirculatory perfusion	Pediatric observational association; therapeutic benefit unproven	Hypoalbuminemia is associated with glycocalyx disruption, increased vascular permeability, and microcirculatory abnormalities in septic children. However, pediatric trials have not established that albumin replacement specifically improves oxygen metabolism, mortality, organ dysfunction, or long-term outcomes.
Inotropes and vasoactive agents	Low cardiac output; impaired oxygen delivery; altered vascular tone	May improve systemic oxygen delivery by increasing cardiac output or optimizing vascular tone according to hemodynamic phenotype	Established component of pediatric septic shock care, but oxygen-metabolism-specific targets remain uncertain	Vasoactive agents are widely used in pediatric septic shock, but optimal drug selection and targets vary by age, cardiac function, and vascular phenotype. Their effects on microcirculatory perfusion and tissue oxygen utilization are heterogeneous and require individualized reassessment.
Vasodilator-based microcirculatory strategies	Hemodynamic incoherence; capillary flow maldistribution	May improve convective and diffusive microcirculatory flow in selected patients with persistent microvascular hypoperfusion despite restored macro-hemodynamics	Physiological rationale; adult-extrapolated and selected observational evidence	Potential benefits are mainly supported by physiological reasoning, adult critical-care experience, and selected experimental or observational studies. Pediatric outcome benefits, safety, timing, and patient selection remain insufficiently defined.
Fresh frozen plasma	Endothelial glycocalyx degradation; vascular permeability	Plasma components may theoretically replenish glycocalyx constituents and support endothelial homeostasis	Mechanistic rationale; insufficient pediatric clinical evidence	FFP has been proposed as an endothelial or glycocalyx-directed intervention, but routine FFP transfusion for improving microcirculatory oxygen metabolism is not supported by high-quality pediatric sepsis evidence. Use should remain guided by conventional indications rather than theoretical glycocalyx repair alone.
Balanced crystalloids	Hyperchloremic acidosis; endothelial stress; fluid-related metabolic derangement	May reduce chloride load, limit hyperchloremic acidosis, and decrease secondary endothelial or renal stress during resuscitation	Pediatric clinical practice with supportive observational and extrapolated evidence	Balanced crystalloids are increasingly favored in resuscitation strategies, but their direct effect on pediatric sepsis oxygen metabolism and microcirculatory outcomes remains incompletely defined. Fluid responsiveness, overload risk, and age-specific physiology should guide use.
Microcirculatory monitoring-guided intervention	Persistent tissue hypoperfusion despite apparently adequate macro-hemodynamics	Direct or indirect assessment of microcirculation may identify hemodynamic incoherence and guide repeated reassessment of perfusion-targeted therapies	Emerging monitoring approach; limited pediatric validation	Sublingual imaging, capillary refill time, perfusion indices, and related tools may help identify microcirculatory dysfunction. However, pediatric reference ranges, feasibility, interobserver reliability, and outcome-linked treatment thresholds require further validation.

**Table 5:** Age-specific considerations for oxygen-metabolism monitoring and management in pediatric sepsis.

Domain of Challenge	Specific Pediatric Manifestations	Clinical Implications	Evidence Gap or Evidence Source	Future Research Directions
Age-specific physiology	Neonates and infants have limited myocardial reserve, immature vascular tone regulation, higher baseline oxygen consumption, and developmental differences in cardiac output distribution	Adult-derived thresholds for ScvO2, lactate interpretation, veno-arterial CO2 difference, and hemodynamic targets may not be directly applicable	Pediatric physiology is well recognized, but age-specific oxygen-metabolism thresholds remain insufficiently validated	Establish age- and weight-specific reference ranges for oxygen-metabolism parameters in neonates, infants, children, and adolescents
Hemodynamic heterogeneity	Children with sepsis may present with low cardiac output, vasodilatory shock, myocardial dysfunction, or mixed phenotypes	A single resuscitation target may be misleading; therapy should be adjusted according to hemodynamic phenotype and reassessment trends	Evidence is derived from pediatric septic shock practice, expert consensus, and limited phenotype-specific studies	Develop phenotype-based pediatric resuscitation algorithms linked to oxygen delivery, perfusion, and clinical outcomes
Microcirculatory monitoring	Capillary refill time, skin temperature, perfusion index, sublingual imaging, and other bedside tools may reflect tissue perfusion, but feasibility varies by age	Persistent microcirculatory dysfunction may be missed when macro-hemodynamic variables appear normalized	Pediatric validation remains limited, especially for device-based microcirculatory monitoring and age-specific normal ranges	Validate microcirculatory monitoring tools against lactate kinetics, organ dysfunction, mortality, and long-term outcomes in pediatric sepsis
Device design and signal quality	Smaller limb circumference, fragile neonatal skin, variable subcutaneous tissue thickness, edema, peripheral vasoconstriction, skin pigmentation, movement, crying, and probe displacement may affect measurements	Sensor contact, optical signal penetration, motion artifacts, calibration accuracy, and perfusion-related measurements may be unreliable in some children	Most wearable or AI-enabled devices have feasibility or signal-quality data, but limited pediatric sepsis outcome validation	Require age-stratified testing of feasibility, safety, signal quality, calibration, and clinical validity before routine use
Evidence base deficit	Many interventions are supported by adult data, small pediatric observational cohorts, animal studies, or mechanistic rationale	Therapeutic claims may be overstated if evidence source is not clearly identified	High-quality pediatric RCTs are scarce for microcirculation-targeted therapy, albumin for glycocalyx protection, FFP for endothelial repair, metabolic modulators, and hypothermia	Conduct multicenter pediatric trials and prospective registries with predefined oxygen-metabolism endpoints
Advanced organ support	ECMO and CRRT are used in selected patients with refractory cardiopulmonary failure, acute kidney injury, fluid overload, or multiorgan dysfunction	Their effect on oxygen metabolism is often indirect and influenced by baseline disease severity	Pediatric evidence supports selected clinical indications, but oxygen-metabolism-specific benefit remains difficult to isolate	Distinguish primary organ-support indications from oxygen-metabolism-targeted effects in future studies
AI and precision monitoring	AI models may integrate lactate trends, ScvO2, perfusion indices, vital signs, wearable signals, and laboratory data	Potential applications include early risk stratification and repeated reassessment, but models may be biased by age, setting, and data quality	Current evidence is mainly feasibility, retrospective modeling, or adult extrapolation; prospective pediatric sepsis validation is limited	Prioritize interpretable, externally validated, age-calibrated AI models with clinically meaningful endpoints

**Table 6:** Adult-derived evidence and pediatric interpretation in oxygen-metabolism management.

Topic	What Is Commonly Derived from Adult or Mixed-Population Evidence	Pediatric Interpretation
Lactate thresholds	Adult sepsis studies often use fixed lactate thresholds for risk stratification and resuscitation assessment	Pediatric interpretation should emphasize serial trends, age group, perfusion signs, hepatic clearance, metabolic reserve, and organ dysfunction
ScvO2 targets	Adult EGDT historically used ScvO2 targets such as >70%	Pediatric ScvO2 should not be treated as a universal target; interpretation depends on age, hemoglobin, cardiac output, sedation, disease phenotype, and oxygen extraction
Microcirculatory imaging	Adult studies support sublingual microcirculation as a marker of hemodynamic incoherence	Pediatric use requires age-specific feasibility, image acquisition standards, reference ranges, and outcome-linked thresholds
Albumin and endothelial glycocalyx	Adult and mechanistic studies suggest links among albumin, glycocalyx integrity, and vascular permeability	Pediatric data support associations but do not establish that albumin replacement improves oxygen metabolism or outcomes
Vasodilator-based strategies	Adult and experimental data suggest possible improvement in microcirculatory flow	Pediatric use should be phenotype-specific, cautious, and outcome-validated
CRRT and ECMO	Adult and mixed critical-care studies inform organ support strategies	Pediatric application depends on age, size, vascular access, circuit volume, anticoagulation, pharmacokinetics, and disease severity
Therapeutic hypothermia	Mechanistic and selected adult data suggest reduced metabolic demand	Pediatric sepsis evidence remains insufficient; use should be investigational or limited to specific indications

## Data Availability

No new data were created or analyzed in this study. Data sharing is not applicable to this article.

## Abbreviations

The following abbreviations are used in this manuscript:

ScvO2	central venous oxygen saturation
ATP	adenosine triphosphate
ROS	reactive oxygen species
AUC	area under the curve
PICU	pediatric intensive care unit
LAR	lactate-to-albumin ratio
DO2	oxygen delivery
VO2	oxygen consumption
RQ	respiratory quotient
EGDT	early goal-directed therapy
FFP	fresh frozen plasma
VV-ECMO	Venovenous extracorporeal membrane oxygenation
PARDS	pediatric acute respiratory distress syndrome
PaO2	arterial oxygen tension
SaO2	oxygen saturation
FiO2	reaction of inspired oxygen
VIS	vasoactive-inotropic score
CRRT	continuous renal replacement therapy
RCTs	randomized controlled trials
AI	artificial intelligence
